# Krackow suturing combined with the suture-bridge technique versus Kirschner-wire tension band combined with patellar cerclage for the treatment of inferior pole patella fracture: a retrospective comparative study

**DOI:** 10.1186/s13018-025-05926-6

**Published:** 2025-05-24

**Authors:** Xujie Yan, Kai Wang, Xueyuan Jia, Yongjun Rui, Ming Zhou

**Affiliations:** 1 Department of Orthopaedic Surgery, Wuxi Ninth People’s Hospital Affiliated to Soochow University, Liangxi Road, No.999, Binhu District, Wuxi, Jiangsu China; 2 Suzhou Medical College of Soochow University, Suzhou, China; 3 Department of Orthopaedic Surgery, Binzhou Medical University Hospital, Binzhou, China

**Keywords:** Krackow suture, Suture-bridge technique, Kirschner-wire tension band, Patellar cerclage

## Abstract

**Background:**

Krackow suturing and suture bridge technique was compared with Kirschner-Wire (K-wire) tension band and patella cerclage for the fixation of inferior pole patella fracture.

**Methods:**

This was a retrospective study of 47 patients with inferior pole patella fractures who underwent fixation procedures at our hospital between January 2019 and May 2022, of whom 25 had Krackow suturing combined with the suture-bridge technique (GROUP 1), and 22 received a K-wire tension band combined with patellar cerclage (GROUP 2). We compared the operative time, reoperation rate, Böstman score, knee range of motion (ROM), fracture-healing time, Insall–Salvati index, complications and hospital expenses between the two groups.

**Results:**

The average follow-up period was 23.1 ± 5.8 months. The complication and reoperation rates in the GROUP 2 were significantly higher than those in the GROUP 1 (*P* = 0.023 and *P* < 0.001). While the GROUP 1 has lower hospital expenses than GROUP 2 (*P* < 0.001). However, significant differences were not found regarding the Böstman score, knee ROM, Insall–Salvati index, fracture-healing time, and operation time between the two groups.

**Conclusions:**

Both Krackow suturing combined with the suture-bridge technique and the K-wire tension band technique can achieve comparable clinical efficacy in stably fixing inferior patellar pole fractures, subsequently allowing an early commencement of rehabilitation exercise, while the suture-bridge technique can also reduce the incidence of complications, hospital expenses and the need for reoperation surgery.

**Trial registration:**

This study was registered on https://www.chictr.org.cn (registration No. ChiCTR2300072069, 2023/06/01, retrospectively registered). Informed consent was obtained from all individual participants included in the study.

## Introduction

Patellar fractures account for about 1% of all fractures, and are commonly caused by the combined effects of direct and indirect trauma, which can subsequently damage the integrity of the knee extension and destroy patellofemoral congruity [1]. As the inferior pole of the patella has no articular cartilage, fractures of the distal quarter are designated a special type of patellar fracture. Although inferior patellar fractures are rare (9.3–22% of all patellar fractures) and often involve small, comminuted fragments, they can significantly impair knee extension [2]. The patella serves as the fulcrum of the knee extension device to provide two moment arms: the quadriceps tendon and the patellar tendon. Tremendous stress is transmitted through the patellofemoral joint. The change of patellar height will lead to the extension of the moment arm of the knee extension device, which will increase the force required for knee extension [3]. Therefore, surgical fixation is preferred in the management of inferior patellar fractures for effectively re-establishing the continuity of the knee extension to expedite the early commencement of functional exercises, toward maximizing restoration of the knee function [4, 5].

Resection of the inferior pole of the patella can lead to patella baja, which would predispose the patient to patellofemoral arthritis affecting the knee function [6–8]. Consequently, surgical fixation of inferior pole patellar fractures has now become the standard of care for this type of injury. Various fixation methods have been reported for surgically treating fractures at the inferior patellar pole, including Kirschner-wire (K-wire) tension band [9], inferior-rim basket plate [10], suture anchor fixation [11], and separate vertical wiring [12]. However, inferior pole patellar fractures are mostly comminuted, so it is difficult to effectively reposition such fractures and fix them using traditional K-wires or screws [13]. Besides, metal internal fixation implants can cause a flare up of irritation reactions in the soft tissues, which can increase the risk of a reoperation being needed, which would thus increase the cost of treatment [14].

In recent years, several studies have advocated the use of non-metallic implants, such as bio-absorbable screws and non-absorbable sutures, for the fixation of patellar fractures [15]. We developed and previously reported a new fixation method using Krackow suturing combined with a suture-bridge technique to treat fractures of the inferior patellar pole, which showed encouraging clinical results in our patients [16]. In the present study, we retrospectively compared and analyzed the clinical efficacy and radiographic outcomes of our new combination technique versus the K-wire tension band technique to determine whether Krackow suturing combined with the suture-bridge technique can safely and effectively fix fractures of the inferior patellar pole. Our hypothesis is that this combined technique yields comparable Böstman score, ROM, fracture-healing time and Insall–Salvati index to the traditional K-wire tension band method.

## Materials and methods

### Patient enrollment

The data from 56 consecutive patients with inferior pole patellar fractures treated surgically at our hospital between January 2019 and May 2022 were retrospectively reviewed. Participants meeting predefined eligibility criteria (inclusion/exclusion) were enrolled. The inclusion criteria were: (1) unilateral inferior pole patellar fracture confirmed by X-ray or CT; (2) fresh and closed fracture; (3) age greater than 18 years old; (4) follow-up period greater than 12 months; and (5) surgery performed by the same surgeon; while the exclusion criteria were: (1) patients with incomplete clinical data; (2) patients with bilateral patellar fractures or unilateral inferior pole patellar fractures combined with other fractures or injuries; (3) patients with other knee disorders; (4) patients with a history of metabolic and/or psychiatric disorders; and (5) patients with severe medical diseases that meant they could not tolerate surgery.

**Fig. 1:**
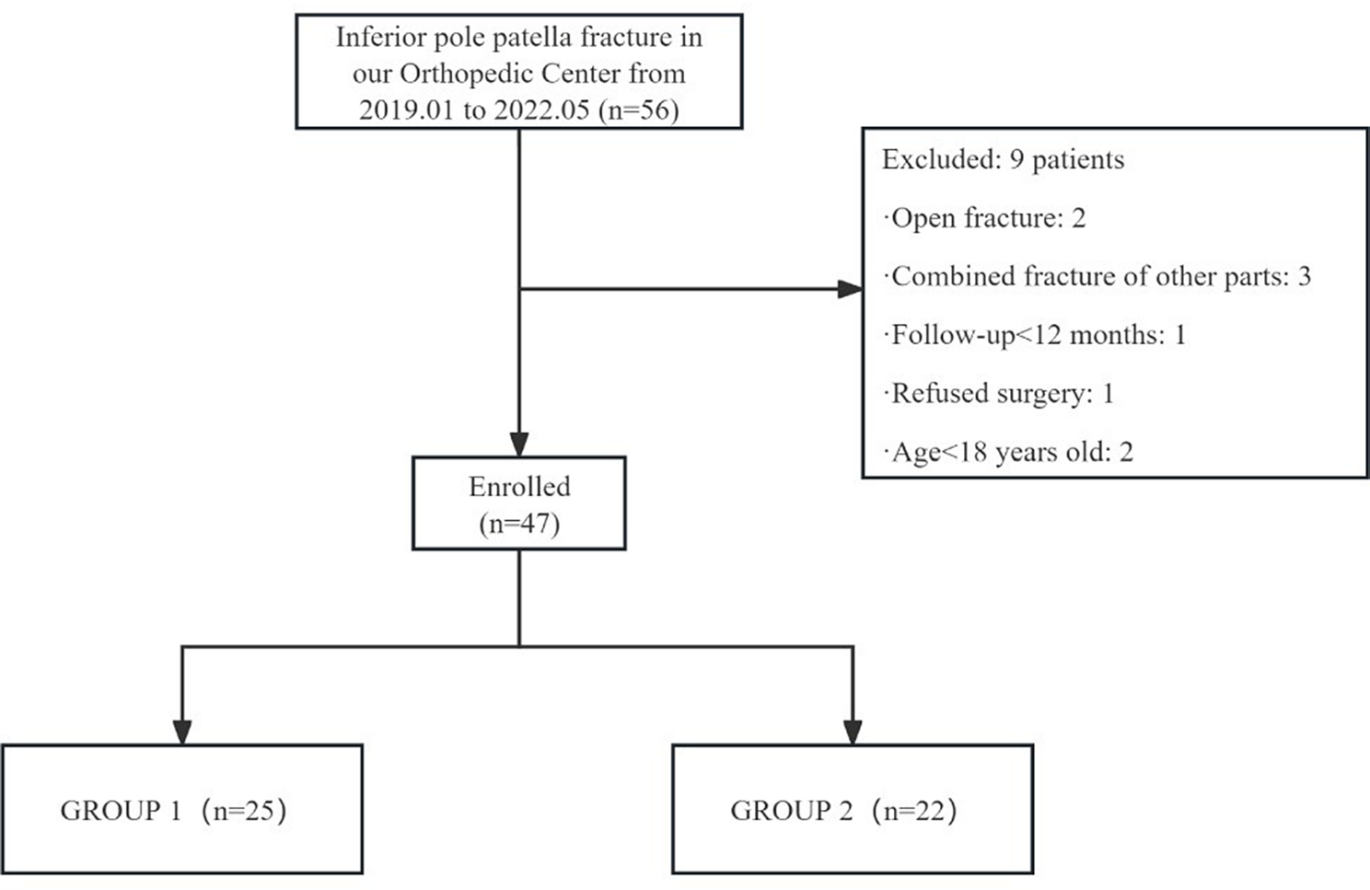
Flow chart of the study for patient inclusion. Group 1: Krackow suturing and suture bridge technique, Group 2: K-wire tension band combined with patellar cerclage technique

After evaluation of the patients in line with the above criteria, 47 patients were included in the study and were divided into two groups: the GROUP 1, comprising 25 patients who had Krackow suturing combined with the suture-bridge technique, and the GROUP 2, comprising 22 patients who had applied the K-wire tension band combined with patellar cerclage (Fig. 1). The choice of surgical technique the patient underwent was arrived at through a collaborative decision-making process between the surgeon and the patient. This decision was preceded and informed by an exhaustive discussion and consideration of the risks and benefits associated with the various treatment options. The study was approved by the Ethics Committee of our hospital (NO: KS2023018), and the study was registered at https://www.chictr.org.cn (registration No. ChiCTR2300072069). All the patients signed an informed consent form. We present the study under the STROBE guidelines [17].

### Surgical technique

**Fig. 2:**
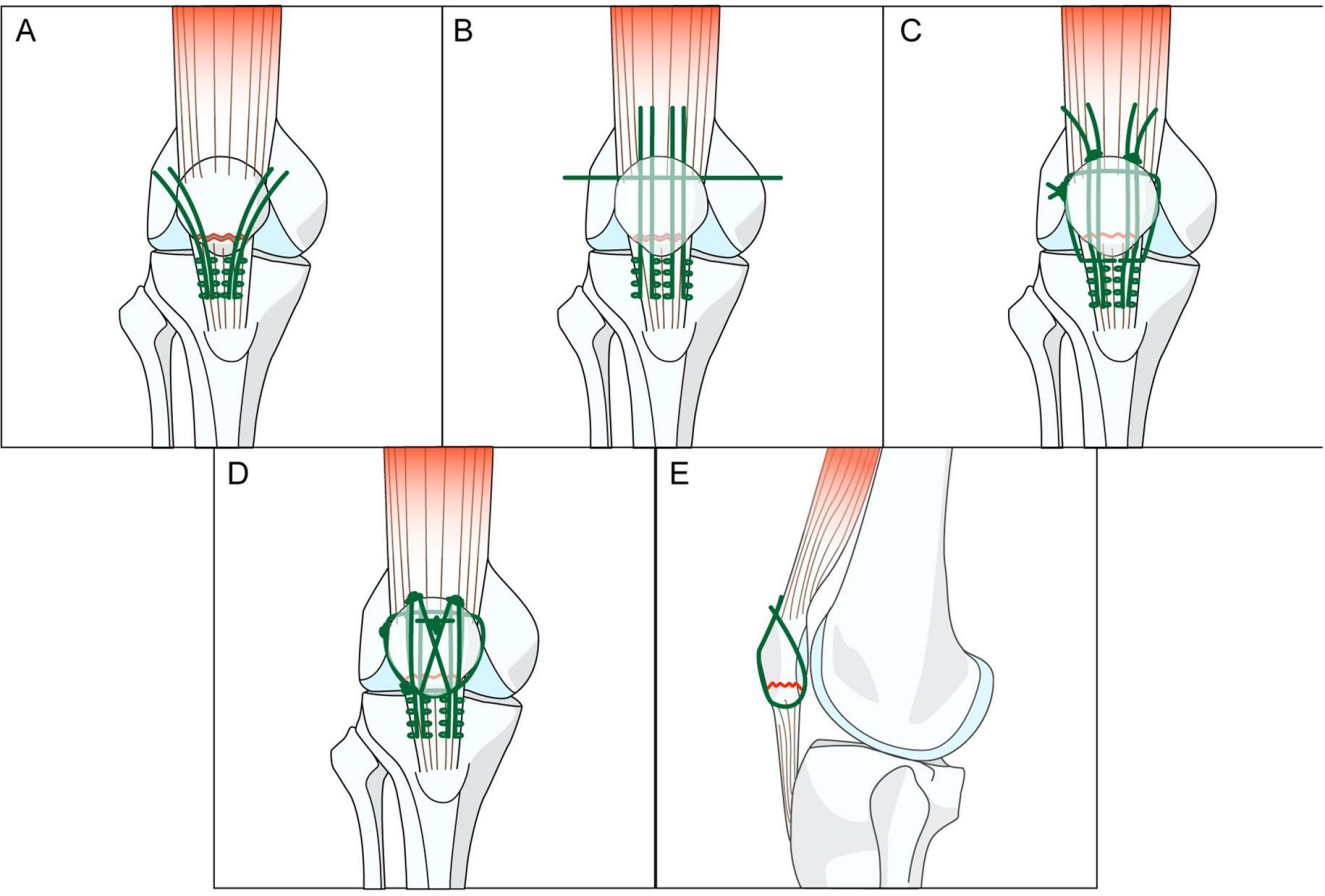
Illustration of Krackow suturing combined with the suture-bridge technique. **A**: Krackow suturing was performed to suture the distal fracture fragments together and attach these to the patellar tendon. **B**: Four longitudinal tunnels were drilled to partition the long axis of the patella into five parts. A tunnel was then transversely drilled through the proximal fracture fragment for passing sutures. **C**: The four free ends of the sutures were passed through the longitudinal tunnel and then tied to the superior pole of the patella. Patellar cerclage was done in the transverse tunnel. **D**: The two free ends of the suture in the middle were crossed in front of the patella and were guided using the reserved sutures toward the superior pole of the patella. The other two free arms of the sutures on both sides were passed in front of the patella to the lower part of the patellar tendon, and were tightened and knotted to form a suture bridge. **E**: Two tunnels were drilled obliquely toward the lower edge of the patellofemoral articular surface 1.5–2.0 cm from the fracture line in front of the proximal fracture fragment, for passing sutures through the lower edge of the distal fracture fragment

**Fig. 3:**
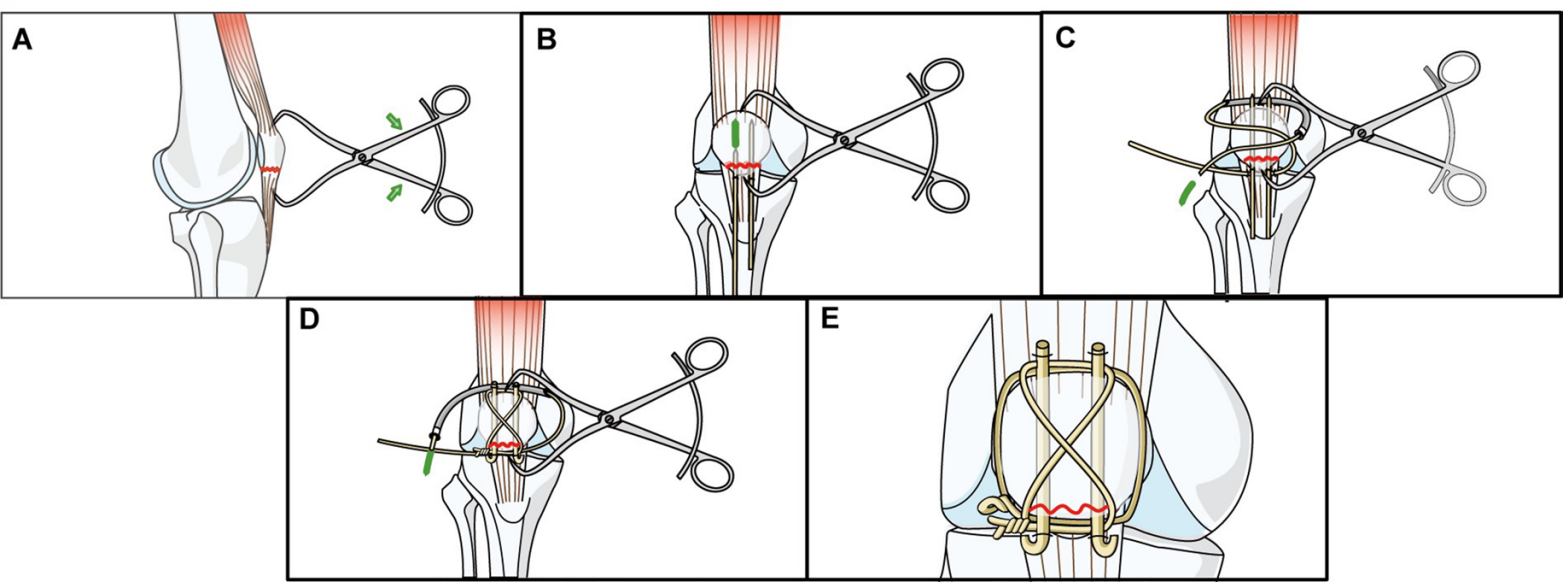
Illustration of the Kirschner-wire tension band combined with patellar cerclage technique. **A**: The patella was reset using Bone tenaculums. **B**: The distal and proximal fracture fragments were fixed using K-wires. **C**: One wire was placed about the protruding K-wire tips for applying figure-of-eight suturing. **D**: Patellar cerclage was performed using another 1.0 mm wire. **E**: The K-wire tension band combined with patellar cerclage

The detailed description of the surgical procedures has been omitted, as the technique has been previously reported [16, 18]. Briefly, for the patients in the GROUP 1, Krackow suturing combined with the suture-bridge technique were employed to stabilize the fracture, along with patellar cerclage and repair of the patellar retinaculum. In our surgical design, we strategically positioned the holes in distinct planes to minimize the risk of confluence. Specifically, we drilled four longitudinal tunnels superficial to the articular surface, one transverse tunnel near the anterior surface, and two obliquely oriented tunnels. (Fig. 2) For the GROUP 2, longitudinal suturing with K-wires and figure-of-eight suturing were performed, followed by patellar cerclage and closure of the incision. (Fig. 3) Intraoperative fluoroscopy was used to confirm the fixation and stability of the repaired fracture in both groups. All surgeries in this study were performed by one senior doctor who has more than 20 years of clinical experience.

### Postoperative management

Based on previous literature [18–20], we employed an identical rehabilitation exercise program for patients who underwent both surgical procedures. Patients began with quadriceps isometric contraction training and ankle mobility training on the second postoperative day. One week later, the knee was trained in passive flexion and extension, with the aim that knee flexion should gradually reach 90° at 4 weeks after surgery and full flexion (flexion > 120°) at 6–8 weeks after surgery. The non-weight-bearing period was one week post-surgery, followed by a gradual transition from partial to full weight-bearing under appropriate protection from weeks 1 to 6 post-operatively, with a knee immobilizer being utilized for the full 6 weeks. At 6 months postoperative, all the patients were able to resume their normal daily activities, but without performing heavy work. For a detailed account of the follow-up visits, please refer to the section titled “Evaluation of the clinical outcomes”.

### Evaluation of the clinical outcomes

All the patients were followed up at 1, 2, 3, 6, and 12 months after the surgical fixations and annually thereafter. Clinical and radiographic improvements were assessed during the follow-up visits. Meanwhile, the hospital expenses of the patients were collected and analyzed. The evaluative parameters of the clinical efficacy included the operative time, reoperation rate, range of motion (ROM) of the knee, Böstman score, and related complications. While the radiological assessment included X-ray images of the fracture healing and the evaluation of Insall–Salvati index. The clinical Böstman grading scale measures the range of movement (3–6 points), pain (0–6 points), work (0–4 points), atrophy (0–4 points), assistance in walking (0–4points), effusion (0–2 points), giving way (0–2 points), and stair-climbing (0–2 points). Functional recovery was evaluated according to the Böstman scores and was classified as excellent (30–28 points), good (27–20 points), and poor (< 20 points) [21]. Insall–Salvati index is measured with the knee flexed 30 degrees, it is the ratio between the patellar tendon length (from the inferior pole of the patella to the tibial tuberosity) and the articular surface length of the patella. The normal range is between 0.8 and 1.2, with patella baja diagnosis if < 0.8 and patella alta diagnosis if > 1.2 [22]. Additionally, the fracture-healing time was recorded. The clinical criteria for confirming fracture healing were blurring of the fracture line and the presence of continuous bone scabs on the fracture line.

To minimize information bias, outcome assessors, comprising radiologists and physical therapists, were blinded to the study group assignments throughout data collection and analysis, and clinical outcomes performed data extraction using standardized case report forms. These assessors had no prior involvement in either the surgical procedures or postoperative care. Patient records were anonymized before analysis, with group assignments coded as ‘Group 1’ and ‘Group 2’. Surgical details, such as implant type, were omitted from the postoperative clinical notes reviewed by the assessors.

### Statistical analysis

Statistical analysis was performed using SPSS for Windows, version 19.0 (SPSS, Inc., Chicago, IL, USA). The countable data were expressed as the mean and standard deviation (SD). The study utilized the chi-square test to analyze and compare the groups for gender, cause of fracture, fracture pattern, reoperation rate, and complications. Additionally, the independent samples t-test was applied to compare the two study groups for age, follow-up (months), Insall–Salvati ratio, and operation time. The Mann–Whitney U-test was employed to compare the two study groups considering the time to operation (d), range of motion at final follow-up, Böstman score, and fracture healing time. A *P*-value < 0.05 was considered statistically significant.

## Results

The study included 47 patients with inferior pole patellar fractures: 25 underwent Krackow suturing with suture-bridge fixation (GROUP 1), and 22 received K-wire tension band with patellar cerclage (GROUP 2). Demographic data were comparable between groups (Table 1).

**Table 1:** Comparison of patient demographics across the study groups

	Group 1(*n* = 25)	Group 2(*n* = 22)	*P*
Gender			0.798^#^
Male	15(60.0%)	14(63.6%)	
Female	10(40.0%)	8(36.4%)	
Age(years)	46.3 ± 9.3	47.5 ± 10.4	0.683^##^
Cause of fracture			0.797^#^
Fall injury	19(76.0%)	16(72.7%)	
Traffic accident injuries	6(24.0%)	6(27.3%)	
Fracture pattern			0.679^#^
Simple	7(28.0%)	5(22.7%)	
Comminution	18(72.0%)	17(77.3%)	
Time to operation (d)	4.1 ± 1.5	4.1 ± 1.6	0.949^###^

Group 1: Krackow suturing and suture bridge technique, Group 2: K-wire tension band combined with patellar cerclage technique. # Based on chi-square test; ## Based on independent samples; ### Based on Mann-Whitney U-test

Clinical outcomes revealed no significant differences in knee range of motion (extension: 1.56 ± 1.26° vs. 2.18 ± 1.37°, *P* = 0.112; flexion: 127.48 ± 4.21° vs. 123.86 ± 9.24°, *P* = 0.085), Böstman scores at 6 months (26.28 ± 2.32 vs. 25.95 ± 2.21, *P* = 0.626) or final follow-up (28.56 ± 1.27 vs. 27.91 ± 1.48, *P* = 0.110), fracture healing time (11.2 ± 1.7 vs. 11.1 ± 1.6 weeks, *P* = 0.898), or Insall–Salvati indices (Table 2). Operative times were similar (55.5 ± 5.1 vs. 53.9 ± 4.7 min, *P* = 0.265).

**Table 2:** Comparison of postoperative outcomes across the study groups

	Group 1(*n* = 25)	Group 2(*n* = 22)	*P*
Follow-up (months)	23.0 ± 6.4	23.3 ± 5.2	0.836^##^
Range of motion at final follow-up (degree°)			
Extension	1.56 ± 1.26	2.18 ± 1.37	0.112^###^
Flexion	127.48 ± 4.21	123.86 ± 9.24	0.085^###^
Böstman score at 6 months after operation	26.28 ± 2.32	25.95 ± 2.21	0.626^###^
Böstman score at final follow-up	28.56 ± 1.27	27.91 ± 1.48	0.110^###^
Fracture healing time (weeks)	11.2 ± 1.7	11.1 ± 1.6	0.898^###^
Insall–Salvati ratio			
Immediate postoperative X-ray	1.01 ± 0.04	1.03 ± 0.05	0.148^##^
Last follow-up X-ray	0.99 ± 0.03	1.02 ± 0.05	0.065^##^
Operation time (minutes)	55.5 ± 5.1	53.9 ± 4.7	0.265^##^
Reoperation rate	0%	72.73%(16 of 22)	< 0.001^#^
Hospital expenses (Chinese Yuan, CNY)	15684.54 ± 801.13	20864.75 ± 3098.55	< 0.001^###^
Complications			
Occasionally giving way	2	1	0.023^#^
Weakness of the quadriceps muscle	1	2	
Symptomatic implants requiring removal	0	4	
Stiffness of the knee	0	1	
Wire break	0	1	

Group 1: Krackow suturing and suture bridge technique, Group 2: K-wire tension band combined with patellar cerclage technique. # Based on chi-square test; ## Based on independent samples; ### Based on Mann-Whitney U-test

**Fig. 4:**
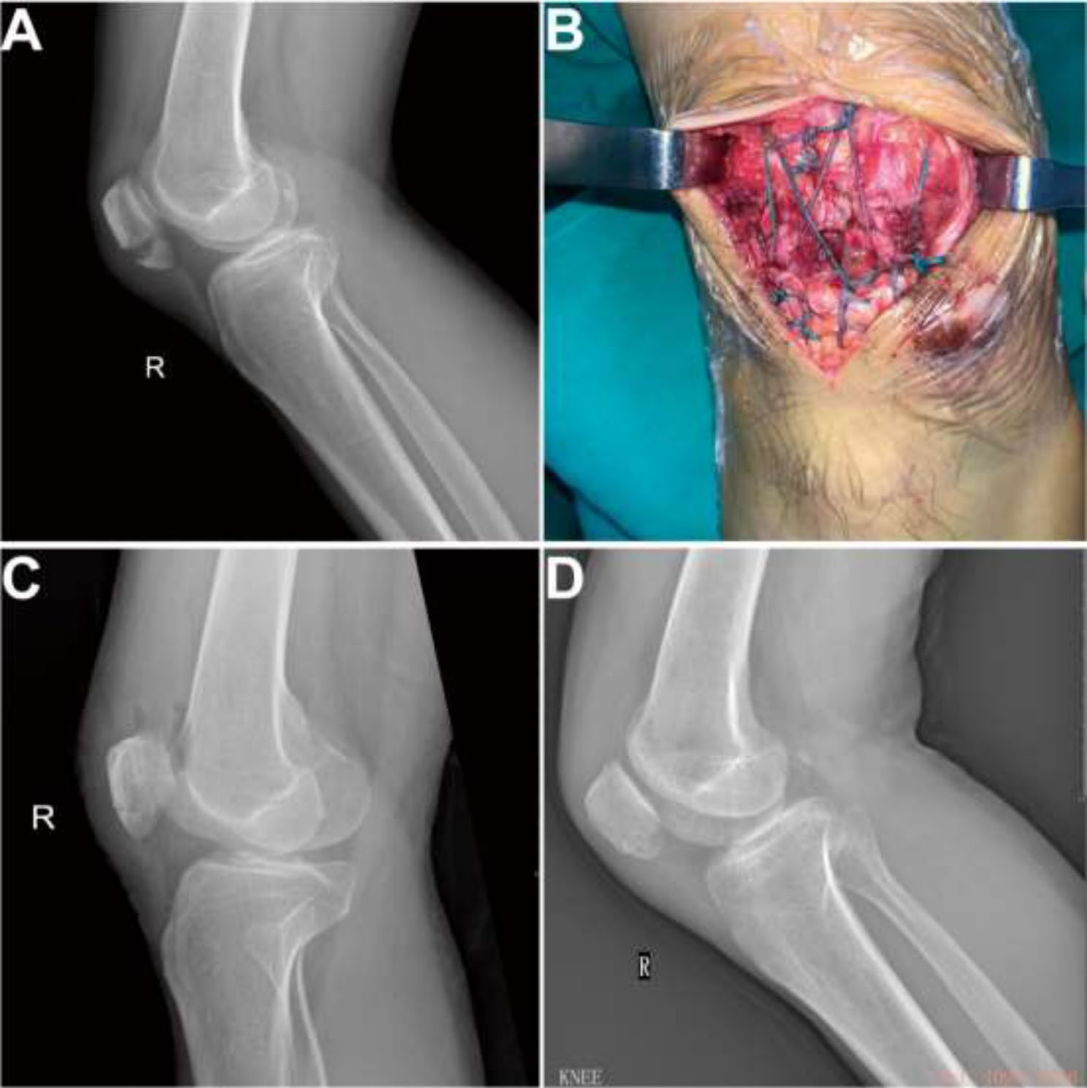
Case vignette No. 1. A 41-year-old man with a simple inferior pole patella fracture (right) from a fall injury with an AO classification of 34-A1 underwent Krackow suturing combined with the suture-bridge procedure. (**A**) Preoperative X-ray presentation; (**B**) Inferior pole patella fracture pulled by Krackow suturing and fixed by a suture bridge via turning over the free arms of the sutures; (**C**) Postoperative X-ray presentations; (**D**) X-ray presentations at 15 months after the operation

**Fig. 5:**
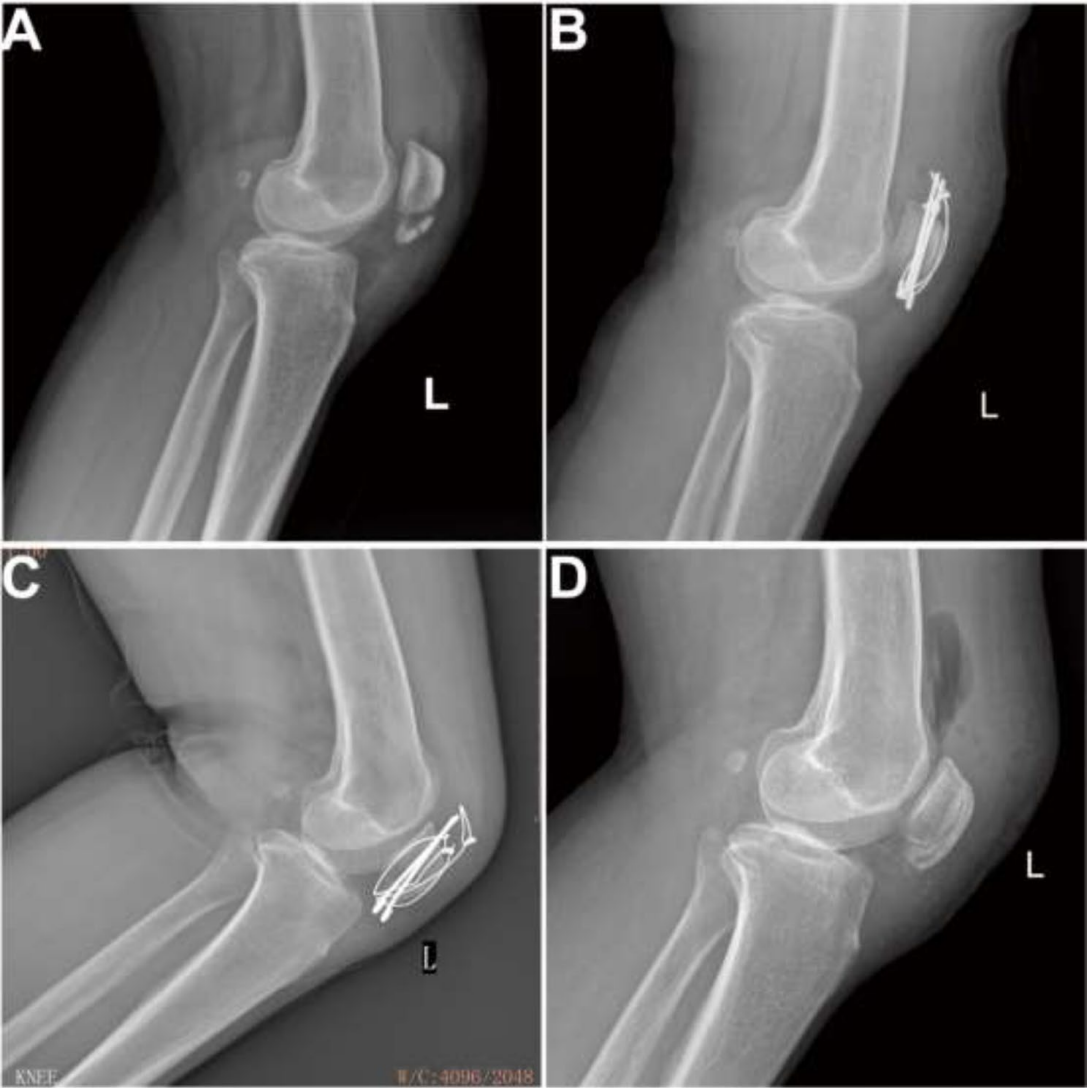
Case vignette No 2. A 52-year-old man with a comminuted inferior pole patella fracture (left) from a fall injury with an AO classification of 34-A1 underwent a Kirschner-wire tension band combined with patellar cerclage technique. (**A**) Preoperative radiographic results. (**B**) Fracture fixed by the Kirschner-wire tension band combined with patellar cerclage technique. (**C**) X-Ray presentations of the broken wire at 13 months after surgery. (**D**) X-Ray image after the implants were removed

GROUP 1 demonstrated significantly lower complication rates (12% vs. 40.9%, *P* = 0.023), no reoperations (0% vs. 72.7%, *P* < 0.001), and reduced hospital costs (15684.54 ± 801.13 CNY vs. 20864.75 ± 3098.55 CNY, *P* < 0.001). In GROUP 1, 3 complications were reported: occasional knee instability (2 cases) and quadriceps weakness (1 case). In GROUP 2, 9 complications were observed: occasional knee instability (1 case), quadriceps weakness (2 cases), symptomatic implant removal (4 cases), knee stiffness (1 case), and wire fracture (1 case). All patients achieved fracture union without infection or arthritis. Some typical cases are shown in Figs. 4 and 5.

## Discussion

Our study confirms that the combination of Krackow suturing and the suture-bridge technique yields clinical outcomes comparable to those achieved with the traditional K-wire tension band for inferior pole patellar fractures, while significantly reducing complications, reoperation rates, and associated costs. Functional recovery metrics, including Böstman scores, knee range of motion (ROM), and fracture healing time, demonstrated no statistically significant differences between the groups (*P* > 0.05). Notably, the suture-bridge group exhibited a substantially lower complication rate (12% vs. 40.9%, *P* = 0.023), eliminated the need for reoperations (0% vs. 72.7%, *P* < 0.001), and reduced hospital costs (15,684 ± 801 vs. 20,865 ± 3,099 CNY, *P* < 0.001). These advantages are likely attributable to the biomechanical superiority of multidirectional suture constructs, which distribute stress more evenly compared to rigid K-wire systems, thereby reducing the risk of implant failure in comminuted fractures [23–25].

Inferior pole patellar fractures pose distinct surgical challenges due to the presence of small, osteopenic fragments and the high tensile forces exerted by the patellar ligament. Traditional surgical approaches, such as partial patellectomy, carry the risk of complications like patella baja and arthritis [26]. Although K-wire tension bands are biomechanically effective for transverse fractures [23], they have inherent limitations when dealing with comminuted fractures. Finite element analyses have demonstrated that stress is concentrated at the K-wire interfaces [25], which predisposes patients to fracture displacement, soft tissue irritation, and even posing a risk of potentially life-threatening complications [27]. These findings are consistent with our results, where 40.9% of patients treated with K-wires developed complications, such as symptomatic implants and wire breakage, and 72.7% required reoperations. This reoperation rate aligns with the 93% removal rate reported by Egol et al. [28].

The suture-bridge technique addresses these challenges by obviating the need for metallic implants. Ethibond sutures offer sustained mechanical strength, comparable to that of metal constructs in the context of transverse patellar fractures, while simultaneously avoiding hardware-related morbidity [29–34]. In our cohort, the suture-bridge technique demonstrated preserved patellar height, as evidenced by the Insall–Salvati index (0.99 ± 0.03 vs. 1.02 ± 0.05, *P* = 0.065), and all patients achieved full knee flexion. This suggests that reduced soft tissue inflammation may facilitate earlier mobilization. Notably, the single instance of knee stiffness in the K-wire group, characterized by flexion of less than 90°, stands in stark contrast to the outcomes observed in the suture group. This implies that non-metallic fixation may better support rehabilitation protocols.

The economic implications further substantiate the advantages of the suture-bridge approach. The 25% reduction in costs (15,684 vs. 20,865 CNY) represents a significant saving, equivalent to 1.5 times the national average monthly income in China (2024: 3,442 CNY/month) [35]. This cost-effectiveness is particularly advantageous in resource-limited settings. These savings are consistent with the findings of Camarda et al., who reported similar cost benefits with FiberWire techniques [15, 32], thereby highlighting suture-based fixation as a cost-effective alternative.

The current study has several limitations that warrant consideration. Firstly, its retrospective design and the collaborative decision-making process involving both surgeons and patients regarding the choice of surgical technique may have introduced selection bias. Patients with financial constraints may have opted for the suture-bridge technique (GROUP 1) due to its lower cost, while those prioritizing perceived stability of metallic implants may have chosen GROUP 2. Additionally, surgeons might have preferentially assigned comminuted fractures to GROUP 1 based on subjective assessments of suture efficacy. Factors such as patient preferences, differences in payment ability, or preoperative counseling could have influenced group assignment, potentially confounding the study outcomes. Although baseline demographics showed no statistical differences, unmeasured confounders such as socioeconomic status or preoperative functional status could have influenced outcomes. Future randomized trials with standardized inclusion criteria are necessary to mitigate this bias. Secondly, as a single-center retrospective cohort study with fewer than 50 patients, and classified as level IV evidence in the hierarchy of clinical evidence, our findings do not permit a definitive conclusion regarding the superiority of one surgical method over the other based solely on the results presented here. Thirdly, the study did not record contralateral patellar height and mobility, which precludes an assessment of whether the length of the patellar ligament and the range of motion of the knee were altered following the fixation surgery. Fourthly, postoperative MRI was not routinely performed to evaluate occult soft tissue injuries; while preoperative clinical and intraoperative evaluations confirmed isolated fractures, advanced imaging in future studies could further elucidate causes of postoperative symptoms like “giving way.” Lastly, while we hypothesize that multidirectional suture constructs reduce stress concentration compared to rigid K-wire systems, the absence of biomechanical testing in this study limits our ability to validate this mechanism. Future biomechanical studies comparing load-to-failure and cyclic loading between these techniques are needed to confirm the structural advantages of the suture-bridge technique.

## Conclusions

Krackow suturing combined with the suture-bridge technique can clinically achieve effective fixation for inferior pole patella fractures with a comparable outcome to the K-wire tension band combined with patellar cerclage technique, subsequently expediting early rehabilitation exercise and reducing the incidence of complications and the need for a reoperation.

## Data Availability

The datasets used and/or analysed during the current study are available from the corresponding author on reasonable request.
